# Diversity of Helminth Parasites in the Fat Sand Rat, *Psammomys obesus* Cretzschmar, 1828, from Tunisia

**DOI:** 10.3390/ani16213492

**Published:** 2026-11-04

**Authors:** Ahlem Boubakri, Hichem Kacem, Slaheddine Selmi, Xavier Roca-Geronès, Jordi Miquel

**Affiliations:** 1 Laboratoire Écologie et Environnement (LR24ES17), Faculté des Sciences de Gabès, Université de Gabès Zrig, Gabès 6072, Tunisia; boubakriahlem8@gmail.com (A.B.); hichem.kacem@fss.usf.tn (H.K.); slaheddine.selmi@fsg.rnu.tn (S.S.); 2 Département des Sciences de la Vie, Faculté des Sciences de Sfax, Université de Sfax, Sfax 3000, Tunisia; 3 Secció de Parasitologia, Departament de Biologia, Sanitat i Medi Ambient, Facultat de Farmàcia i Ciències de l’Alimentació, Universitat de Barcelona, Av. Joan XXIII, sn, 08028 Barcelona, Spain; xavierroca@ub.edu; 4 Institut de Recerca de la Biodiversitat (IRBio), Universitat de Barcelona, Av. Diagonal, 645, 08028 Barcelona, Spain

**Keywords:** helminth fauna, *Gerbillitaenia psammomi*, *Sudarikovina aegyptica*, *Mesocestoides* sp. larvae, *Syphacia* sp., infection parameters, host–parasite interactions

## Abstract

*Psammomys obesus* is a diurnal herbivorous rodent widely distributed across the arid regions of North Africa and the Arabian Peninsula. Although it has ecological, agricultural, public health, and biomedical importance, its helminth fauna remains poorly known. This study provides a comprehensive inventory of helminth parasites infecting *P. obesus* in southern Tunisia and examines variations in parasitism according to host sex, age, body condition, and sampling region. A total of 152 specimens of *P. obesus* were captured between September 2023 and March 2026 in the Tamaghza and Djebel Dahar regions. Parasitological examination revealed four helminth species: three cestodes, *Gerbillitaenia psammomi*, *Sudarikovina aegyptica*, and *Mesocestoides* sp. larvae, and one nematode, *Syphacia* sp. Their respective prevalences were 63.8%, 12.5%, 0.7%, and 16.4%. The species are morphologically described, and genetic data and a phylogenetic tree are provided for *Syphacia* sp. The findings of *Mesocestoides* and *Syphacia* species represent new records for *P. obesus*. Furthermore, *S. aegyptica* constitutes a new record for Tunisia. Host age and sampling region significantly influenced the occurrence and abundance of *G. psammomi*, as adults were more likely to be infected and harbored a significantly higher parasite burden than juveniles. In contrast, neither host sex nor body condition was significantly associated with parasite occurrence or abundance. No significant association between parasite occurrence or abundance and the host’s sex, age, or body condition was found for *S. aegyptica* and *Syphacia* sp.

## Simple Summary

The helminthological study of 152 fat sand rats (*Psammomys obesus*) from two regions in southern Tunisia (Tamaghza and Djebel Dahar) has recovered four parasite species: three cestodes (*Gerbillitaenia psammomi*, *Sudarikovina aegyptica* and *Mesocestoides* sp.) and one nematode (*Syphacia* sp.). *Mesocestoides* and *Syphacia* species are new records for *P. obesus*, and *S. aegyptica* constitutes a new record for Tunisia. This study evaluated the influence of host sex, age, body condition, and sampling region on parasitic parameters (occurrence and abundance).

## 1. Introduction

The fat sand rat (*Psammomys obesus*) is a diurnal rodent widely distributed throughout the arid and semi-arid regions of North Africa, from Mauritania to Egypt and Sudan, with its range extending eastward into the Arabian Peninsula [1,2]. It primarily inhabits dense and complex burrow systems excavated in sandy substrates, generally beneath shrubs such as *Haloxylon alicornicum*, a member of the Chenopodiaceae family. These plant formations provide the species with protection, particularly against avian predators [3,4,5,6,7].

From an ecological perspective, *P. obesus* plays an important role in the functioning of arid terrestrial ecosystems. It serves as prey for several predators, including snakes, owls, diurnal raptors, and golden jackals, in the trophic web [8]. This species is also widely used in biomedical research as an experimental model of metformin-sensitive nutritional type 2 diabetes and several associated complications, including cataracts, pancreatic atrophy, and impaired renal function [9].

However, *P. obesus* may also have adverse implications for public health and agriculture. Regarding public health, this species is an important reservoir of several vector-borne pathogens, particularly *Leishmania major*, the causative agent of zoonotic cutaneous leishmaniasis [10,11,12,13]. *Psammomys obesus* is also hunted and consumed in some localities of southern Tunisia, representing a potential route of human exposure to parasitic agents, particularly those with zoonotic potential. Regarding agriculture, this rodent’s burrowing activity may damage crops, as well as earthen dikes used for rainwater harvesting and retention, locally known as “Tabias”. Moreover, its habitats are frequently located near agricultural lands and grazing areas. This proximity promotes ecological interfaces between wildlife, domestic animals, human populations, and their shared environment. From a One Health perspective, these interactions emphasize the importance of investigating *P. obesus*’s parasite fauna. Characterizing its parasite diversity and identifying species with zoonotic potential could provide a better understanding of this rodent’s potential role in the maintenance and circulation of parasitic agents within southern Tunisian ecosystems. These data could also provide useful baseline information to better assess potential health risks associated with interactions between wildlife, domestic animals, human populations, and their environment.

Over the past few decades, numerous studies have focused on the role of *P. obesus* in the transmission cycle of *L. major* in arid areas of the Middle East and North Africa [14,15,16]. In contrast, knowledge of the helminth fauna of the fat sand rat remains limited and scattered. The earliest reports of helminths in *P. obesus* mainly originated from parasitological surveys conducted from the mid-twentieth century onwards on rodents inhabiting Saharan regions [17,18,19]. These studies revealed a helminth fauna composed primarily of nematodes and cestodes [20,21,22,23,24,25,26,27,28,29,30,31,32,33,34,35,36].

Furthermore, the ecological and environmental drivers of the occurrence and abundance of helminths associated with *P. obesus* remain poorly investigated, even though such data are essential for gaining a better understanding of their potential role in the occurrence and dynamics of co-infections with other parasites.

This study aimed to investigate the helminth fauna of *P. obesus* in southern Tunisia by determining the prevalence, mean abundance, and mean intensity of the helminth parasites infecting this host. In addition, this study assessed the effects of host sex, age class, sampling site, and body condition on these parasitological parameters.

## 2. Materials and Methods

### 2.1. Host and Parasite Identification

This study was conducted in the Djebel Dahar and Tamaghza areas of southern Tunisia (Figure 1). Between September 2023 and March 2026, we carried out 21 field trips, each lasting between one and nine days, to capture animals and collect samples. Seven trips were successful, resulting in the capture of a total of 152 individuals: 52 in Dahar and 100 in Tamaghza (Table 1).

Fat sand rats were trapped with the permission of the “Direction Générale des Forêts (Ministère de l’Agriculture, des Resources Hydrauliques et de la Pêche Maritime)” of Tunisia (references: 1822/2023 (2 October 2023), 1728/2024 (30 September 2024), 437/25 (13 July 2025), and 50/2026 (15 January 2026)). The protocol for rodent collection and study was approved by the Biomedical Ethics Committee of the Pasteur Institute of Tunis (Document reference: 2021/29/I) on 2 March 2023 as per EU Directive 2010/63/EU.

The animals were captured using snap traps placed at burrow entrances. Traps were set either after individuals had been directly observed returning to their burrows at sunrise or at burrows showing signs of recent activity, such as freshly displaced sand outside the burrow or cut fragments of *Haloxylon salicornicum* near the entrance.

The captured individuals were euthanized by cervical dislocation in the field laboratory and then weighed to the nearest 0.1 g using a pan balance. The total body length, including the head, was measured using a digital caliper to the nearest 0.1 mm. Sex was determined via macroscopic examination of the gonads following dissection. The sample comprised 81 females and 71 males. Age was estimated using a combination of craniometric measurements and the assessment of dental wear patterns. Two distinct age classes were distinguished: young individuals are generally characterized by smaller cranial dimensions and by upper and lower third molars that are either unerupted or only recently erupted, showing little or no wear. Their molars display well-defined cusps, laminae, and labial cusplets, with high enamel relief and limited dentine exposure. In contrast, adults generally exhibit larger cranial dimensions. Their third molars are fully erupted, and dental wear is more pronounced, resulting in a progressive reduction in enamel relief, greater dentine exposure, and, in older individuals, partial coalescence of some laminae and cusps, as well as a loss of definition of the labial cusplets. Thus, cranial size, the eruption status of the third molars, and the degree of dental wear constitute relevant criteria for distinguishing juvenile from adult individuals [37].

The body cavity and internal organs were thoroughly examined for helminths under a stereomicroscope. Recovered platyhelminths were gently flattened between a microscope slide and coverslip and fixed in either 70% ethanol or Bouin’s solution in the field laboratory. Later, once at the university, the specimens were stained with Semichon’s acetocarmine, dehydrated via a graded ethanol series, cleared in clove oil, and permanently mounted in Canada balsam. Nematodes were cleared in Amann’s lactophenol before morphological examination. Helminth species were identified per the relevant literature [25,28,32,33,38,39,40,41,42].

Type specimens of the studied helminths were deposited in the “Museu de Ciències Naturals de Barcelona” (Spain) and in the “Muséum National d’Histoire Naturelle de Paris” (France) (see descriptions of the species and in the Data Availability Statement). *Psammomys obesus* specimens (skull and carcass) were deposited in the “Museu de Ciències Naturals de Barcelona” (Spain) and in the “Museu de Ciències Naturals de Granollers” (Spain) (see Data Availability Statement).

### 2.2. Scanning Electron Microscopy

Some specimens of *Syphacia* sp. were preserved for SEM analysis. Thus, they were fixed in 70% ethanol in the field and subsequently dehydrated via a graded ethanol series (from 80% to absolute ethanol) in the laboratory, followed by critical-point drying with carbon dioxide in an Emitech K850X (Quorum Technologies Ltd., Laughton, East Sussex, UK). The dried worms were then mounted on stubs using conductive adhesive tape, coated with carbon in an Emitech K950X evaporator (Quorum Technologies Ltd.), and examined with a JSM-7001F field-emission scanning electron microscope (JEOL Ltd., Tokyo, Japan) operated at 10 kV at the “Centres Científics i Tecnològics de la Universitat de Barcelona (CCiTUB)”.

### 2.3. Molecular Analysis

A subsample of some *Syphacia* sp. specimens was selected for the molecular analysis. Genomic DNA was isolated using the Pure PCR Template Extraction Kit (Roche Diagnostics S.L., Sant Cugat del Vallès, Spain), according to the manufacturer’s protocol. PCR amplification of the entire ITS region of the rDNA was performed using NC5 and NC2 primers [43], with an annealing temperature of 55 °C. To verify PCR amplification, the product was electrophoresed at 120 V on a 2% agarose gel and visualized under UV light. The amplified products were prepared for sequencing using the same primers as those used for PCR amplification. Sequencing was performed by the Genomics Unit of the “Centres Científics i Tècnics de la Universitat de Barcelona (CCiTUB)”.

No DNA extraction was performed on *Mesocestoides* specimens due to the unavailability of 70% molecular biology-grade ethanol during the fieldwork. Consequently, commercially available 70% ethanol, which was not suitable for molecular analysis, was used for specimen fixation. All larvae fixed in this manner were stained and mounted for morphological examination.

### 2.4. Sequencing Analysis

Sequences were aligned using the Clustal X software (version 2.1) and adjusted manually [44]. Phylogenetic analysis of ITS and 28S rDNA regions examined in this study, along with other species previously deposited in GenBank, was carried out via Bayesian analysis (BI) using the MrBayes software, version 3.2.7 [45]. The sequences used for the analysis are reported in Table S1. The best-fit substitution models were determined by the JModeltest software (version 2.1.10) using the Akaike Information Criterion (AIC) [46]. The Bayesian posterior probability analysis was carried out with 1,000,000 generations and a subsampling frequency of 100. Posterior probabilities were estimated and used to assess support for each branch, considering a well-supported value of 0.90.

### 2.5. Infection Parameters Analysis

Infection parameters, including prevalence, mean abundance, and mean intensity, were calculated for each parasite species following the definitions proposed by Bush et al. [47].

Generalized linear mixed models (GLMMs) were then used to assess the relationships between host sex (two classes: male = 0; female = 1), age [two classes: juveniles (1–2 years old) = 0 vs. adults (>2 years old) = 1], and body condition as fixed effects on the occurrence probability and abundance of recorded helminth species. The sampling period (seven classes) was considered a random factor to account for potential non-independence between observations collected in the same period. Body condition was assessed with the scaled mass index (SMI), following the procedure proposed by Peig and Green [48]. This index provides an estimation of the predicted body mass of an individual when the structural body size of that individual is standardized to a reference value: SMI = M × (L/L_0_)^βSMA^, where M is the body mass of the individual concerned, L is a linear body measurement (body length in our study), and L_0_ is a reference value of L (the mean body length of the studied sample). The scaling exponent β_SMA_ corresponds to the estimated slope of a log–log-standardized major axis (SMA) regression of M on L [48].

Because of the particular data structure, different GLMMs were conducted for the different species recorded. One species occurred in the two regions sampled (see the Results), and thus, we also entered region (Dahar vs. Tamaghza) as a further fixed effect to assess whether the occurrence probability and abundance of this species differed between these two areas. However, this was not performed for the other species as they only occurred in one region each. Occurrence was always modeled by considering a binomial distribution and a logit link function. Abundance was modeled using a negative binomial distribution and a log link function, assuming aggregated parasite distributions among host individuals (high variance-to-mean ratios; see the Results). However, the model did not converge for one species; therefore, the data were log(x+1)-transformed, and we ran a GLMM assuming a normal distribution and an identity link function.

The results are reported as parameter estimates with their associated standard errors (β ± SE), test statistics, and *p*-values. Effects were considered statistically significant at *p* < 0.05. All statistical analyses were performed using the GLIMMIX procedure in the SAS/STAT software, version 9.2 [49].

## 3. Results

Four helminth species were identified: three cestodes, *Gerbillitaenia psammomi* (Mikhail & Fahmy, 1968) (Catenotaeniidae), *Sudarikovina aegyptica* (Mikhail & Fahmy, 1968) (Hymenolepididae), and *tetrathyridium* larvae of the genus *Mesocestoides* (Mesocestoididae), and one nematode, *Syphacia* sp. (Oxyuridae). The four species exhibited distinct geographical distributions. *Gerbillitaenia psammomi* was recorded in Djebel Dahar and Tamaghza, whereas *S. aegyptica* was detected exclusively in Djebel Dahar and *Syphacia* sp. exclusively in Tamaghza. *Mesocestoides* sp. was found only in one fat sand rat from Djebel Dahar (Table 2).

### 3.1. Gerbillitaenia psammomi (35 Specimens Measured; Range, Mean in Parentheses)

Type material: Four specimens were deposited in the “Museu de Ciències Naturals de Barcelona” (Spain); accession numbers: MZB 2024-3090 to MZB 2024-3093. Two specimens were deposited in the “Muséum National d’Histoire Naturelle de Paris” (France); accession number: MNHN HEL2089.

Partial 28S rDNA sequences were deposited in GenBank; accession numbers: PP291541, PP291542, and PP291543.

*Gerbillitaenia psammomi* exhibits 4–36 (16.2) acraspedote proglottids. The strobila measures 5.7–87.0 mm (35.9 mm). The maximum width, at the level of mature or pre-gravid proglottids, is 2.2–4.8 mm (3.3 mm). The anterior end is quite rounded (Figure 2a,b). It has no neck. The scolex is unarmed and measures 578–877 μm (715.8 μm) in width. It has four suckers, more or less round, with a maximum diameter of 206–288 μm (252.5 μm) (Figure 2a,b). The genital primordium appears in the first proglottid, just after the scolex. Mature proglottids are wider than long (Figure 2a,b). The first mature proglottid measures 751–1656 × 2019–4840 μm (1036.0 × 3212.0 μm). The genital pores, in general, alternate regularly and are located near the anterior limit of the proglottid (Figure 2a,b). Mature proglottids contain approximately 221–414 testes (282.2) arranged in two lateral fields; there are 98–202 (129.9) poral testes and 118–212 (152.4) antiporal testes (Figure 2b). The cirrus pouch measures 141–444 × 54–83 μm (264.9 × 69.0 μm). In the center of the proglottid, there is a dendritic, highly branched ovary (Figure 2b). There is no internal or external seminal vesicle. The seminal receptacle is oval, thick-walled, and measures 103–279 × 52–134 μm (185.9 × 81.8 μm) (Figure 2b). The vagina runs parallel to the vas deferens. The vitelline gland is strongly lobed and placed on the poral side pre-equatorially between the seminal receptacle and ovary (Figure 2b). Gravid proglottids are longer than wide, especially the last one, which measures 2640–6212 × 1113–3209 μm (4057.8 × 2136.8 μm). The uterus has a long longitudinal trunk with 6–10 (7.8) primary uterine branches on each side of the longitudinal trunk (Figure 2a). The posterior uterine branches form a prominent fan-like group without a distinct trunk. The eggs, observed after lacerating gravid proglottids, are pear-shaped to fusiform (Figure 2c).

### 3.2. Sudarikovina aegyptica (19 Specimens Measured; Range, Mean in Parentheses)

Type material: Two specimens were deposited in the “Museu de Ciències Naturals de Barcelona” (Spain) under the following accession numbers: MZB 2025-7284 and MZB 2025-7285. One specimen was deposited in the “Muséum National d’Histoire Naturelle de Paris” (France); accession number: MNHN HEL2088.

*Sudarikovina aegyptica* is a small- to medium-sized hymenolepidid cestode. The strobila measures 7.46–39.67 mm (16.67 mm) in length and 279–1063 µm (688.3 µm) in width and consists of 38–132 (72) proglottids. Its globular scolex, which lacks a rostellum and has a diameter of 258–423 µm (331.6 µm), bears four suckers with a maximum diameter of 103–175 µm (123.4 µm) (Figure 3a). The neck measures 283–774 µm (516.1 µm) in length. The acraspedote segments are wider than long, measuring 196–351 µm (268.9 µm) in length and 279–1063 µm (707.9 µm) in width. The genital pores are unilateral. There are 7–10 (8.4) testes: 2–4 (2.9) poral testes and 4–7 (5.5) aporal testes (Figure 3b). The cirrus sac measures 231–370 µm (311.4 µm) in length and 57–103 µm (78.9 µm) in width and encloses an internal seminal vesicle measuring 41–100 × 46–154 µm (63.5 × 87.0 µm) (Figure 3c). An external seminal vesicle measuring 39–100 × 46–141 µm sits close to the cirrus sac (60.7 × 76.0 µm) (Figure 2c). The ovary—measuring 57–90 × 90–103 µm (72.3 × 95.8 µm)—is lobed and situated on the poral side, though slightly offset toward the center of the segment (Figure 3b). The seminal receptacle, located near the external seminal vesicle, measures 57–90 × 90–103 µm (46.9 × 50.1 µm) (Figure 3c). The post-ovarian vitelline gland measures 44–82 × 49–100 µm (54.8 × 65.1 µm). The uterus’s reticular morphology is visible in pre-gravid proglottids (Figure 3d).

### 3.3. Mesocestoides *sp.* larvae

We collected *tetrathyridium* larvae belonging to the genus *Mesocestoides* from a *P. obesus* captured in Ksar Hallouf. These larvae feature an excretory apparatus formed by two collectors that end in an excretory bladder, which opens into an excretory pore. At the opposite pole of the larva, we observe the scolex, often invaginated, without hooks and presenting four suckers (Figure 4).

### 3.4. Syphacia *sp.*

Type material: Thirty-five males and 35 females were deposited in the “Museu de Ciències Naturals de Barcelona” (Spain) under the following accession numbers: MZB 2026-0443 to MZB 2026-0451.

ITS rDNA partial sequences were deposited in GenBank under the following accession numbers: QB130523 and QB130524.

The nematodes are small, particularly the males. The cephalic extremity bears three lips: two lateroventral and one dorsal. There are four cephalic papillae and two amphidial pores. The lateroventral lips each have one cephalic papilla and one amphid, whereas the dorsal lip presents two cephalic papillae. In our specimens, the cephalic papillae are situated close to the amphids.

Male (35 specimens measured; range, mean in parentheses)

The male measures 1000–1437 µm (1208.3 µm) in length, 98–175 µm (134.6 µm) in maximum width, and 77–118 µm (90.2 µm) in width at the level of the esophageal bulb. It is ventrally curved and bears three ventral cuticular swellings aligned along the ventral midline, positioned as follows: the first at 291–586 µm (454.3 µm), the second at 399–753 µm (604.5 µm), and the third at 545–975 µm (813.7 µm) from the anterior extremity (Figure 5a,b). The distance between the amphidial pores in the male is 13–25 µm (19.7 µm). The nerve ring is located 72–113 µm (99.8 µm) from the anterior extremity. The excretory pore is situated 231–432 µm (345.1 µm) from the anterior extremity. The esophagus measures 175–257 µm (228.3 µm) in length, with an esophageal bulb with a diameter of 49–69 µm (58.3 µm) (Figure 5b). The spicule is slender and measures 57–77 µm (68.1 µm) in length. The gubernaculum is fusiform, 28–44 µm (34.1 µm) long, and presents an accessory hook (Figure 5c). Three pairs of caudal papillae are observed: the first pair is preanal, the second is adanal, and the third is postanal and laterally placed (Figure 5d). The tail measures 77–111 µm (91.8 µm). The body ends in a conical process—the caudal tip—measuring 39–67 µm (52.3 µm).

Female (35 specimens measured; range, mean in parentheses)

The body length is 3044–3962 µm (3396.1 µm). The maximum body width is 198–301 µm (254.0 µm); the width is 100–183 µm (142.9 µm) at the level of the esophageal bulb, 180–278 µm (233.9 µm) at the level of the vulva, and 51–90 µm (67.7 µm) at the level of the anus. The esophagus measures 303–386 µm (344.6 µm), and the esophageal bulb has a diameter of 80–111 µm (92.9 µm). The nerve ring is located 100–164 µm (124.4 µm) from the anterior end. The distance between amphidial pores in the female is 28–37 µm (32.3 µm) (Figure 6b). The excretory pore is situated at 334–622 µm (506.2 µm) from the anterior end (Figure 6a). The vulva is located in the anterior third of the body, at 481–985 µm (714.2 µm) from the anterior end (Figure 6a). The tail is very elongated, measuring 301–496 µm (370.3 µm). The eggs are fusiform, asymmetrical, and operculate, measuring 85–111 × 31–43 µm (101.3 × 34.9 µm). The operculum is located on the concave side, near one of the poles (Figure 6c). The eggs are unembryonated at laying.

All ITS rDNA sequences obtained for *Syphacia* specimens were identical and were deposited in GenBank under the accession numbers QB130523 and QB130524. Considering the obtained *Syphacia* sequences, the best-fit substitution model was TIM1+G (G = 0.4251). The Bayesian inference phylogenetic tree based on *Syphacia* ITS sequences revealed high support for all inferred clades, as shown in Figure 7. *Syphacia* sp. obtained in this study formed a distinct lineage and was grouped within the same major clade as *Syphacia obvelata*, *Syphacia nigeriana*, and *Syphacia frederici* (Figure 7).

### 3.5. Influence of Sex, Age, and Sampling Area

The results of the generalized linear mixed models (GLMMs) showed that host age and sampling region significantly influenced the occurrence and abundance of *G. psammomi* in *P. obesus*. In contrast, neither host sex nor the scaled mass index (SMI) showed a statistically significant association with parasite burden at the 5% significance level.

Adult *P. obesus* had a significantly higher probability of harboring *G. psammomi* than juveniles. More specifically, the odds of occurrence were approximately 2.55 times higher in adults than in juveniles. Sampling region also had a significant effect: individuals collected in Djebel Dahar had approximately 4.10 times higher odds of harboring *G. psammomi* than those collected in Tamaghza.

Host age also had a highly significant effect on parasite abundance. The expected abundance of *G. psammomi* was approximately 3.55 times higher in adults than in juveniles. Similarly, abundance was significantly higher in Djebel Dahar than in Tamaghza, with an expected abundance approximately 2.80 times greater in Djebel Dahar (Table 3, Figure 8).

In contrast, host sex had no statistically significant effect on either the occurrence or abundance of *G. psammomi*. The SMI was also not significantly associated with parasite occurrence. Although its estimated effect on abundance was positive, it remained non-significant (Table 3, Figure 8).

For *S. aegyptica*, sex, age, and scaled mass index (SMI) had no statistically significant effect on either the probability of occurrence or abundance (Table 3).

For *Syphacia* sp., sex, age, and the standardized mass index (SMI) of *P. obesus* had no significant effect on either the probability of occurrence or parasite abundance (Table 3). Although females and adults showed slightly higher estimated probabilities of infestation, and SMI was negatively associated with occurrence, none of these trends were statistically significant. Similarly, females showed slightly lower parasite abundance than males, whereas adults showed slightly higher abundance than juveniles. SMI was also negatively associated with parasite abundance, but none of these effects reached statistical significance.

## 4. Discussion

To date, several nematode species have been recorded in *P. obesus*. In Tunisia, Bernard [20,21] reported the presence of *Trichuris muris psammomysi*, *Longistriata seurati*, *Trichosomoides crassicauda*, and *Gongylonema brevispiculum*. *Gongylonema neoplasticum* was subsequently reported in the same country by Jrijer et al. [22]. A representative of the genus *Trichostrongylus* was identified in Israel by Wertheim and Durette-Desset [23], whereas *Trichuris muris* was recorded in Egypt by Anwar et al. [24]. The cestode fauna of *P. obesus* also includes several species reported from different geographical regions. *Sudarikovina aegyptica* was recorded in Egypt by Mikhail and Fahmy [25], while *Pseudocatenotaenia matovi* was reported in Syria by Schmidt et al. [26]. *Gerbillitaenia psammomi* has been documented in several studies conducted in Egypt [27,28,29,30,31,32] and, recently, in Tunisia [33]. In addition, *Raillietina trapezoides* was recorded in Egypt, Palestine, and Tunisia [24,34,35]. Finally, the hymenolepidid *Rodentolepis nana* was reported in captive individuals [36].

Thus, this study constitutes the first finding of *S. aegyptica* in Tunisia. Furthermore, the findings of *Mesocestoides* sp. and *Syphacia* sp. represent new records infecting *P. obesus*. Concerning cestodes, previous studies reported the catenotaeniid *G. psammomi* and the hymenolepidid *S. aegyptica* parasitizing the fat sand rat [25,27,28,29,30,31,32,33]. In addition, the catenotaeniid was also found parasitizing *Meriones shawi* in Morocco [50].

In a previous study, Boubakri et al. [33] performed molecular analysis of *G. psammomi*. The phylogenetic tree based on the 28S rDNA sequences showed two different clades grouping species of Skrjabinotaeniinae and Catenotaeniinae subfamilies. *Gerbillitaenia psammomi* was grouped in the Skrjabinotaeniinae clade together with *Skrjabinotaenia* and *Meggittina* species. According to these authors [33], the observed genetic divergence between *G. psammomi* and *Skrjabinotaenia oranensis* sequences was substantial enough to support their classification as distinct species, corroborating previous morphological distinctions [32,51]. Maximum likelihood analyses further reinforced this separation, with strong nodal support [33]. The creation of the genus *Gerbillitaenia* by Haukisalmy and Elmahy [32] is also supported by the genetic divergence between these two species [33].

Regarding the role of the fat sand rat as a potential intermediate host in maintaining the life cycle of *Mesocestoides*, our results (only one parasitized host) must be considered with caution. However, it might be one of the species previously cited in Tunisia, with various carnivores acting as definitive hosts. In this context, adult stages of three species of the genus *Mesocestoides*, namely, *Mesocestoides corti*, *Mesocestoides lineatus,* and *Mesocestoides litteratus,* have been reported in Tunisia, among which *M. lineatus* is the most frequently reported due to its presence in dogs and cats [52,53,54,55]. In other studies, Lahmar et al. [56,57] reported species of *Mesocestoides* such as *M. lineatus* and *M. litteratus* in rural and stray dogs. Regarding other wild carnivores, *M. corti*, *M. lineatus*, and *M. litteratus* were reported in the red fox (*Vulpes vulpes*) and the golden jackal (*Canis aureus*) with high prevalences, particularly for *M. lineatus* at 22% (*M. corti*); 55.6% (*M. lineatus*) and 33% (*M. litteratus*) in the red fox; and 12.9% (*M. corti*), 74% (*M. lineatus*), and 23% (*M. litteratus*) in the golden jackal [58]. From a health perspective, two species of this genus, namely, *M. lineatus* and *M. variabilis*, have been reported in humans, most of them from the USA and East Asia (Japan, China, and South Korea) [59]. Outside these geographical areas, *M. variabilis* has been reported in a Greenlander living in Denmark, and a case attributed to an unidentified *Mesocestoides* species occurred in Rwanda-Urundi [60,61,62]. All of them are regarded as foodborne zoonoses caused by the consumption of tetrathyridia in undercooked meat, particularly snake blood and liver [59].

Regarding the oxyurid of the genus *Syphacia*, this work represents the first report of this genus in *P. obesus*. In Tunisia, *Syphacia obvelata* has been reported in various Gerbillinae rodents (*Dipodillus campestris*, *Gerbillus gerbillus*, *Gerbillus pyramidum*, *Meriones shawi*, and *Jaculus jaculus*) [21]. However, the author stated that this species was synonymous with *Syphacia nigeriana*. Subsequently, Hugot [40] considered the findings in Tunisian gerbillines to belong to the species *S. nigeriana*. The *Syphacia* found parasitizing the fat sand rat in our study shows certain similarities to *S. nigeriana,* such as the distance between amphids, the egg size in females, and other non-discriminant characteristics, although the spicule in males is smaller in our specimens (57–77 µm vs. 70–95 µm) [37,39,63].

Based on the ITS rDNA-region sequences, the phylogenetic tree generated in the present study recovered *Syphacia* sp. from the fat sand rat in Tunisia within a well-supported major clade that also included *S. obvelata*, *S. nigeriana*, and *Syphacia frederici*, suggesting a close phylogenetic relationship and a common evolutionary origin between these species. A similar phylogenetic arrangement was observed in other studies, which grouped the latter three species within the same major clade [64,65,66]. However, the presence of *Syphacia* sp. from this study in a well-supported subclade suggests its distinct genetic identity within a separate lineage. The association of *Syphacia* sp. with *P. obesus* may, therefore, reflect a distinct host–parasite relationship within this evolutionary lineage, as other members of the major phylogenetic clade are typically associated with different rodent genera, such as *Mus*, *Microtus*, and *Apodemus* [64,65,66]. In this sense, using additional genetic markers in the phylogenetic analysis, such as the mtDNA COI gene, would help clarify the precise phylogenetic placement of *Syphacia* sp.

The prevalence and abundance of *G. psammomi* were significantly higher in adult rodents than in juveniles. This difference may be due to the longer exposure period experienced by adult individuals, which promotes the gradual accumulation of infections throughout their lifetime [67,68]. In addition, adults generally occupy larger home ranges while searching for food or mates, thereby increasing their likelihood of coming into contact with the parasite’s intermediate hosts. Although the life cycle of *G. psammomi* remains to be clarified, these intermediate hosts may include the mite *Glyciphagus domesticus*, as reported for certain species of the family Catenotaeniidae, particularly *Catenotaenia pusilla* [69,70,71].

Furthermore, *P. obesus* specimens captured in Djebel Dahar exhibited a significantly higher occurrence and abundance of *G. psammomi* than those collected in Tamaghza. The ecological mechanisms underlying this geographical difference could not be identified due to our study’s correlational nature, but at least one explanatory hypothesis can be proposed: local ecological or epidemiological conditions may have been more favorable to parasite transmission in Djebel Dahar, such as a greater availability of potential intermediate hosts or environmental conditions conducive to their development and survival. This interpretation remains speculative and should be tested in further studies incorporating data on environmental conditions and the abundance of potential intermediate hosts.

In contrast, neither sex nor body condition appeared to be a major determinant of infection in *P. obesus* in this study. The absence of sex differences may be because males and females share broadly similar habitats, behaviors, and diets, resulting in comparable levels of exposure to the parasite. The lack of a clear trend in the relationship between parasitic infestation and body condition highlights that this relationship is often dynamic, as different, even opposing, trends can occur simultaneously within the same population. For example, a low parasite load might allow the host to make better use of its energy resources and maintain good body condition, thereby increasing its defenses against other parasites. Meanwhile, a host in good condition would be active and occupy a large home range, making it more susceptible to encountering parasites. It would also possess the necessary resources to tolerate a high parasite load and survive [72,73].

*Sudarikovina aegyptica* appears to have a restricted biogeographical distribution, confined to the arid regions inhabited by its definitive host, *P. obesus*. This hymenolepidid cestode was first reported in *P. obesus* in Egypt by Mikhail and Fahmy [25]. In this study, we document *S. aegyptica* for the first time in the fat sand rat in Tunisia, specifically in the Djebel Dahar region. This finding, therefore, extends the known geographical range of the cestode.

The absence of *S. aegyptica* from the Tamaghza region may be associated with the absence or limited availability of suitable intermediate hosts. These hosts may include numerous arthropods, such as coleopters and oribatid mites, which have been identified as intermediate hosts for other members of the family Hymenolepididae, particularly species belonging to the genus *Hymenolepis* [74,75].

The lack of significant effects of sex, age, and body mass index on the prevalence and abundance of *S. aegyptica* suggests that infection may be influenced more strongly by environmental exposure than by the individual characteristics of *P. obesus*. Breeding pairs and their offspring generally share the same burrow and display similar feeding habits, with no marked dietary differences between the sexes. Their diet consists primarily of succulent halophytic plants, particularly species traditionally assigned to the family Chenopodiaceae [1,2,76]. This shared use of habitat and food resources may expose individuals to similar levels of contact with potential intermediate hosts, such as oribatid mites, and may, consequently, explain the comparable infection levels observed between the different host categories.

## 5. Conclusions

The helminth community of *P. obesus* in southern Tunisia consists of three cestodes (*G. psammomi*, *S. aegyptica*, and *Mesocestoides* sp.) and one nematode (*Syphacia* sp.). The occurrence of *Mesocestoides* sp. and *Syphacia* sp. represents new host records for *P. obesus*, while *S. aegyptica* has been reported for the first time in Tunisia. The phylogenetic tree based on rDNA ITS region sequences showed *Syphacia* sp. clustering with the most similar species but situated in a distinct subclade, indicating its distinct genetic identity.

Sampling region and host age significantly influenced the occurrence and abundance of *G. psammomi*. For *S. aegyptica* and *Syphacia* sp., no significant associations were detected between parasite occurrence or abundance and the host characteristics examined. These findings indicate that the determinants of helminth infection in *P. obesus* are parasite-specific and may reflect differences in transmission pathways, host–parasite interactions, and local ecological conditions. Further studies incorporating larger sample sizes, seasonal variation, habitat characteristics, and the availability of intermediate hosts would help to clarify the ecological factors underlying the spatial distribution and transmission dynamics of these helminths.

## Figures and Tables

**Figure 1:**
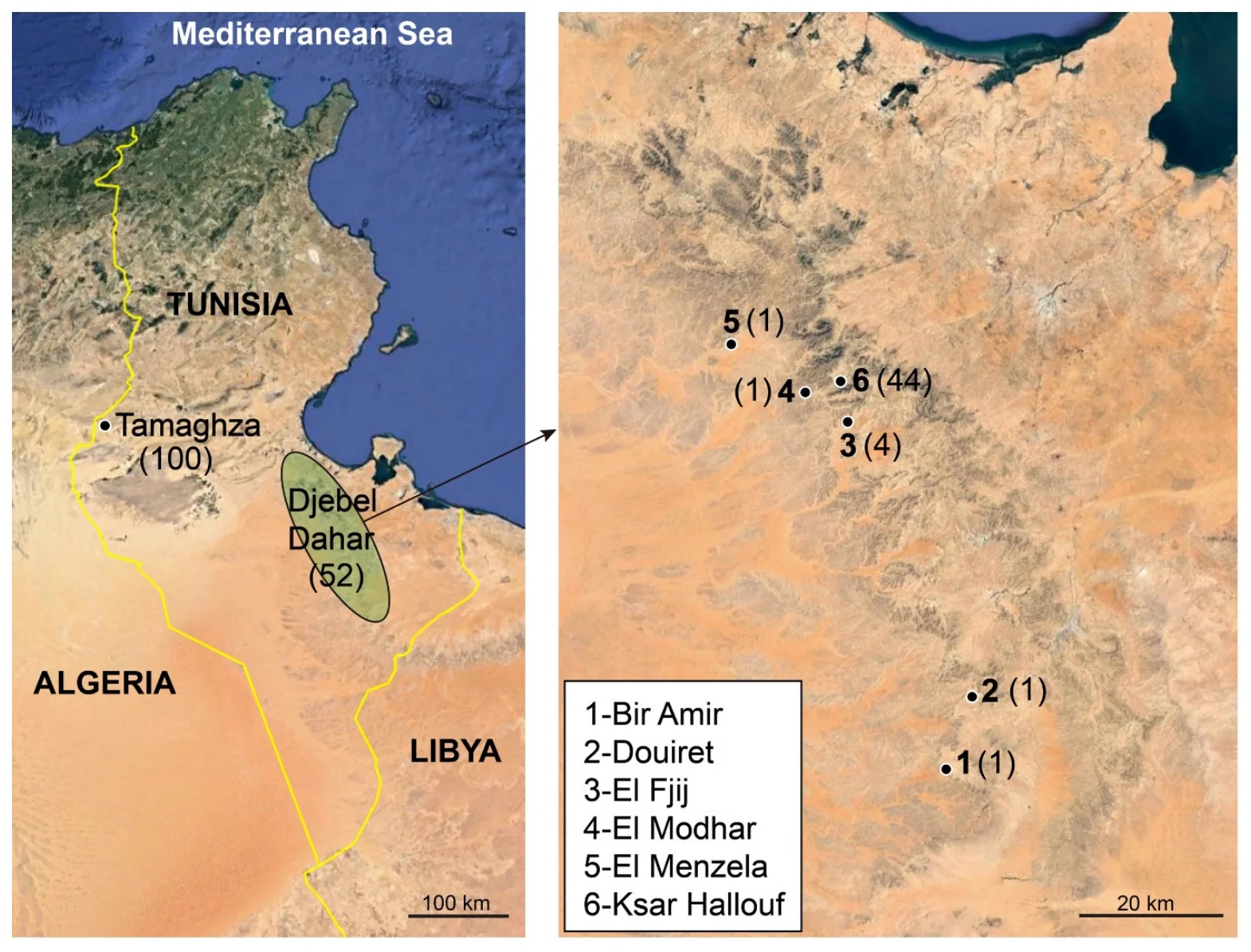
Geographical distribution of the *Psammomys obesus* sampling sites in the Djebel Dahar and Tamaghza regions of Tunisia. The number of specimens captured at each location is shown in parentheses. The images were obtained using Google Earth Pro and subsequently edited with Adobe Illustrator, version 29.8.7 (Adobe, San Jose, CA, USA).

**Figure 2:**
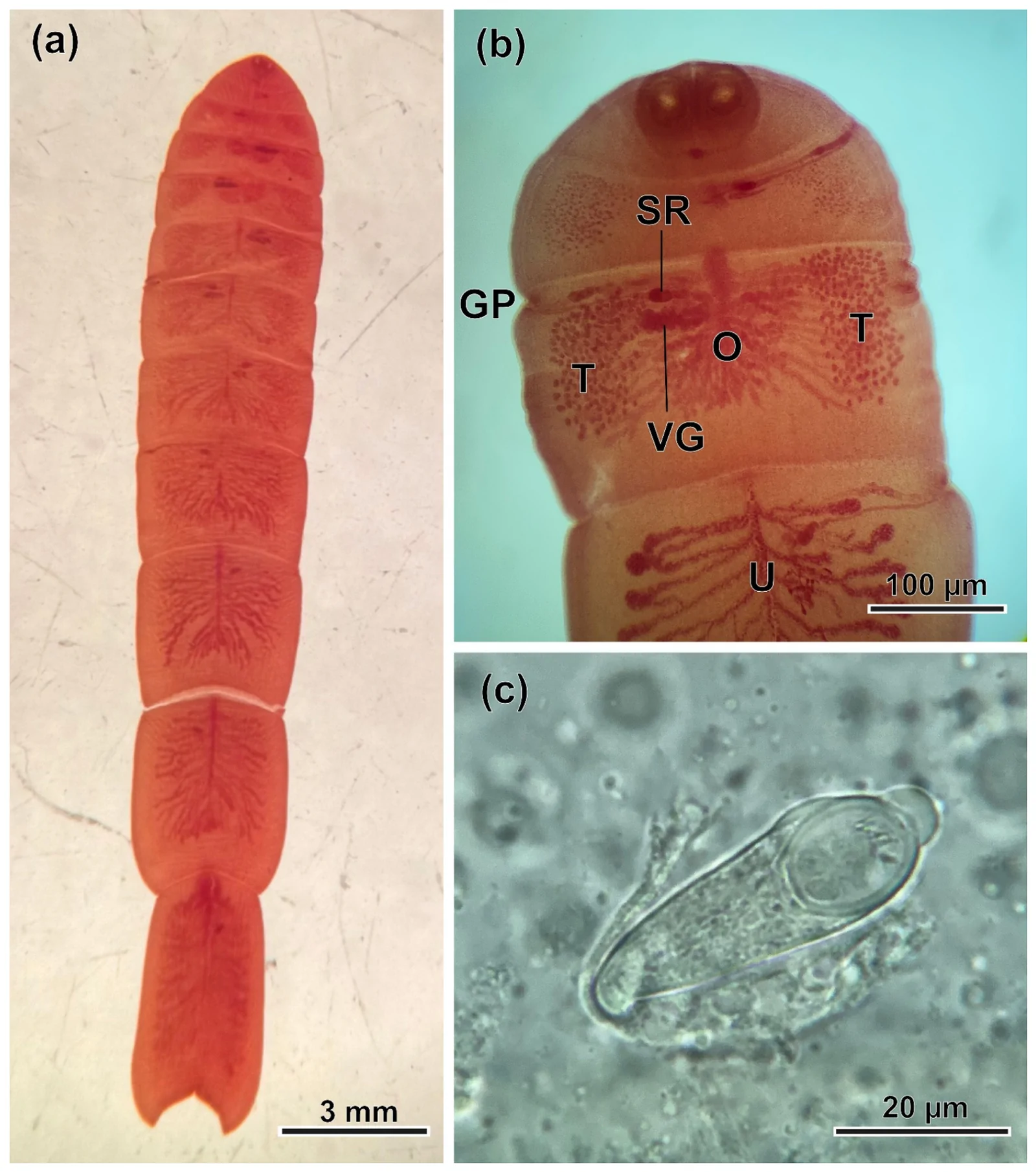
*Gerbillitaenia psammomi*. (**a**) Entire worm. (**b**) Detail of anterior extremity showing scolex and mature proglottid. (**c**) Egg. GP, genital pore; O, ovary; SR, seminal receptacle; T, testes; VG, vitelline gland.

**Figure 3:**
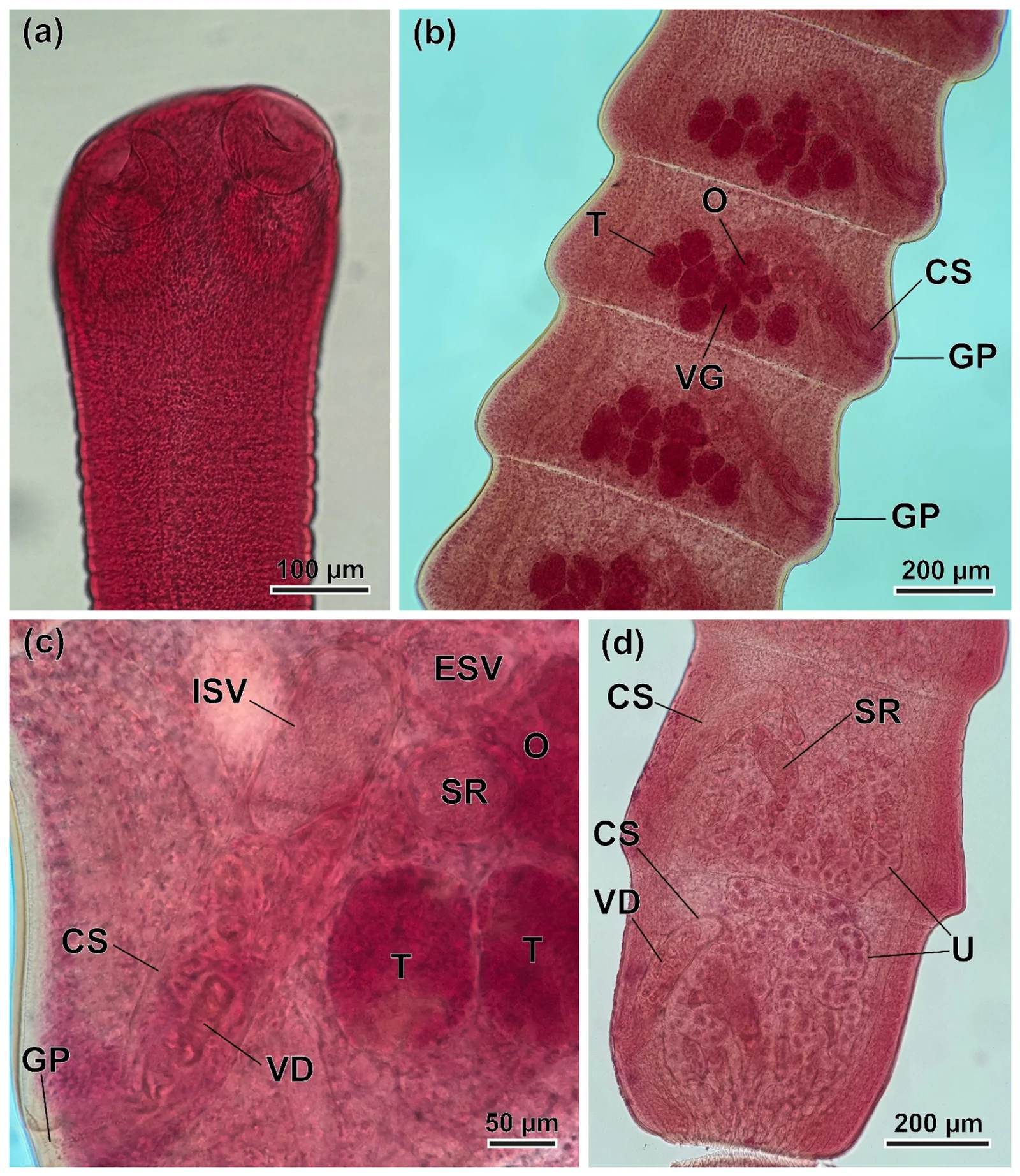
*Sudarikovina aegyptica*. (**a**) Scolex. (**b**) Mature proglottids. (**c**) Detail of mature proglottid near the genital pore (GP). (**d**) Pregravid proglottid showing the reticular uterus (U). CS, cirrus sac; ESV, external seminal vesicle; ISV, internal seminal vesicle; O, ovary; SR, seminal receptacle; T, testes; VD, vas deferens; VG, vitelline gland.

**Figure 4:**
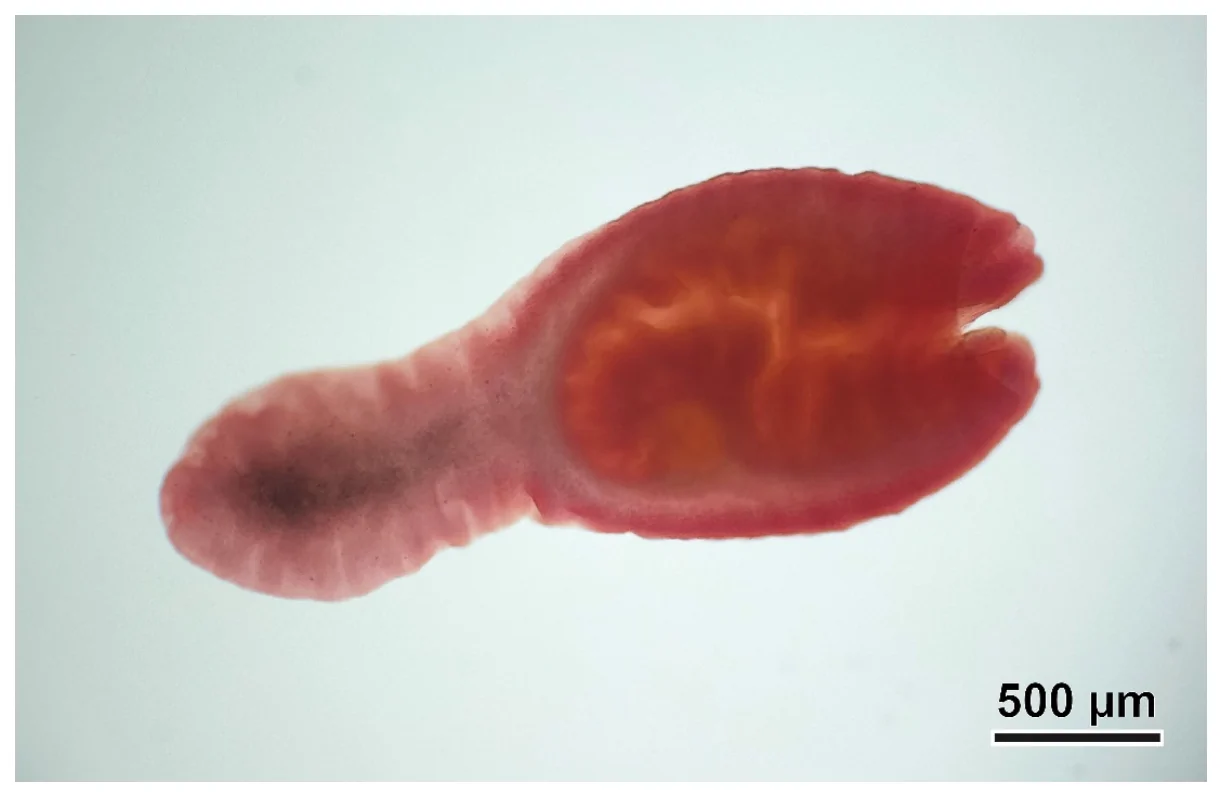
*Mesocestoides* sp., *tetrathyridium* larvae.

**Figure 5:**
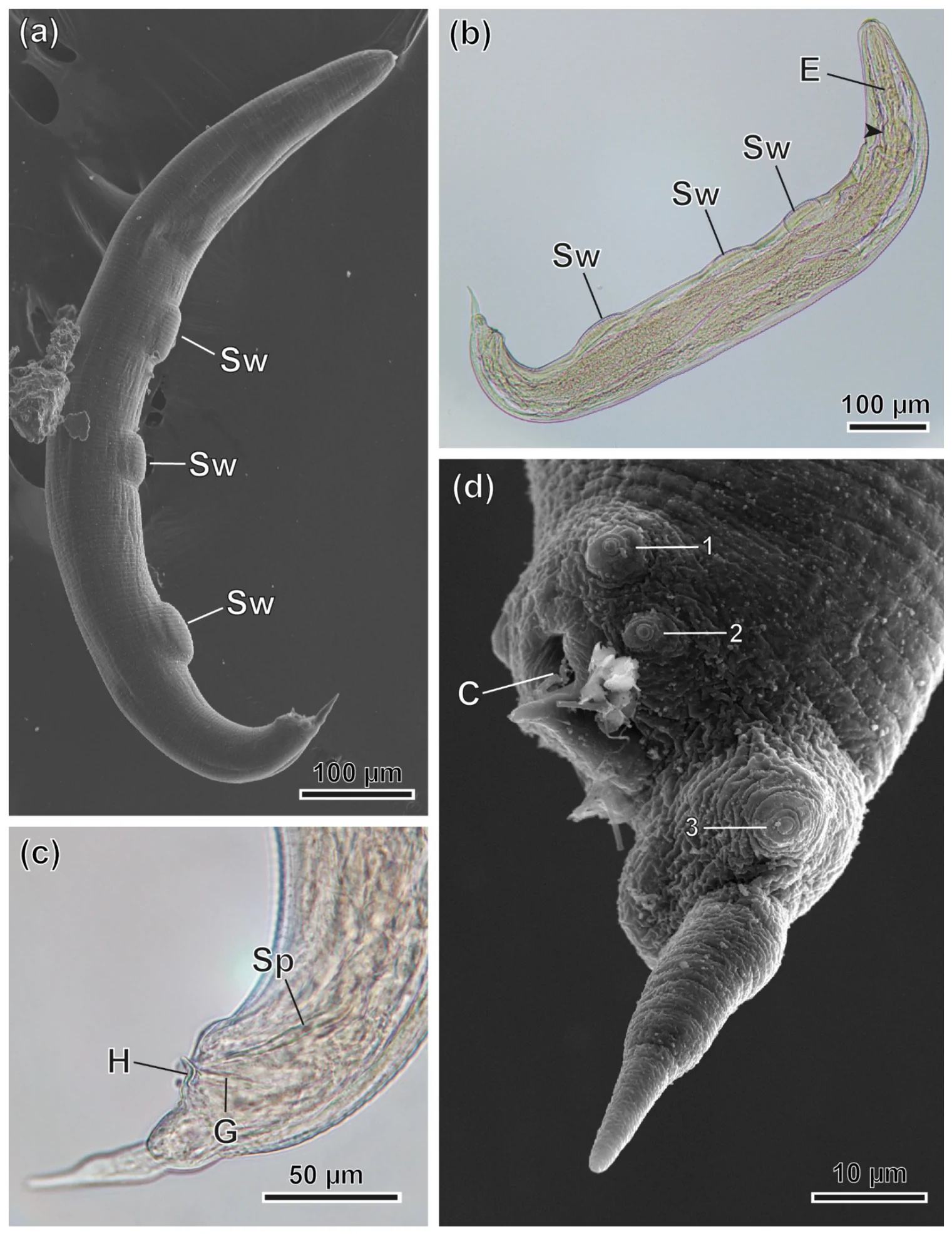
*Syphacia* sp., male, LM and SEM. (**a**,**b**) Entire worms in LM and SEM showing the three cuticular swellings (Sw). (**c**) Caudal extremity in LM showing the spicule (Sp), the gubernaculum (G), and the hook of the gubernaculum (H). (**d**) Caudal extremity in SEM showing the three pairs of caudal papillae (1–3). Arrowhead, esophageal bulb; C, cloaca; E, esophagus.

**Figure 6:**
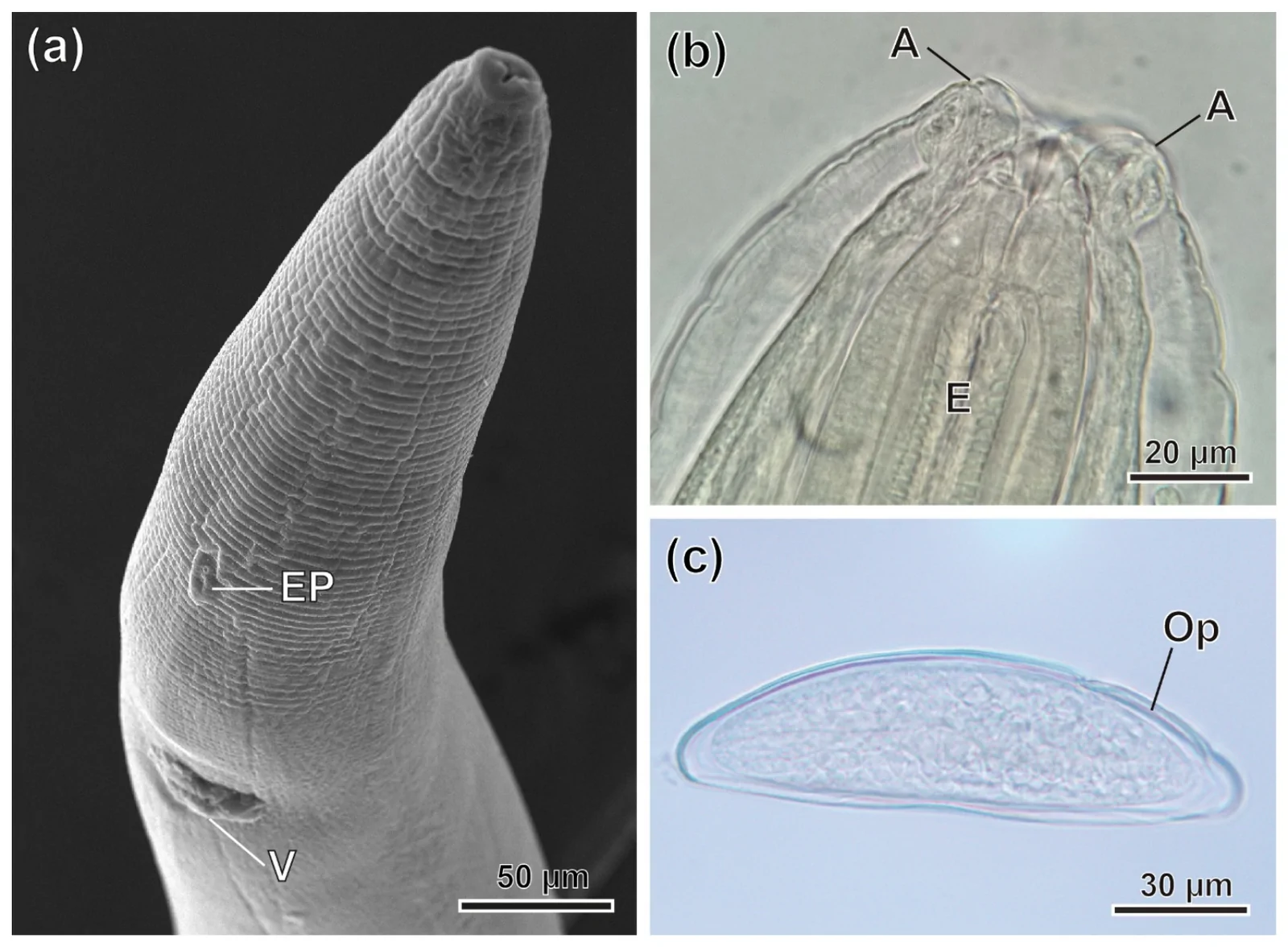
*Syphacia* sp., female, LM and SEM. (**a**) Anterior extremity in SEM showing the excretory pore (EP) and the vulva (V). (**b**) Detail showing the amphidial pores (A). (**c**) Egg. E, esophagus; Op, operculum.

**Figure 7:**
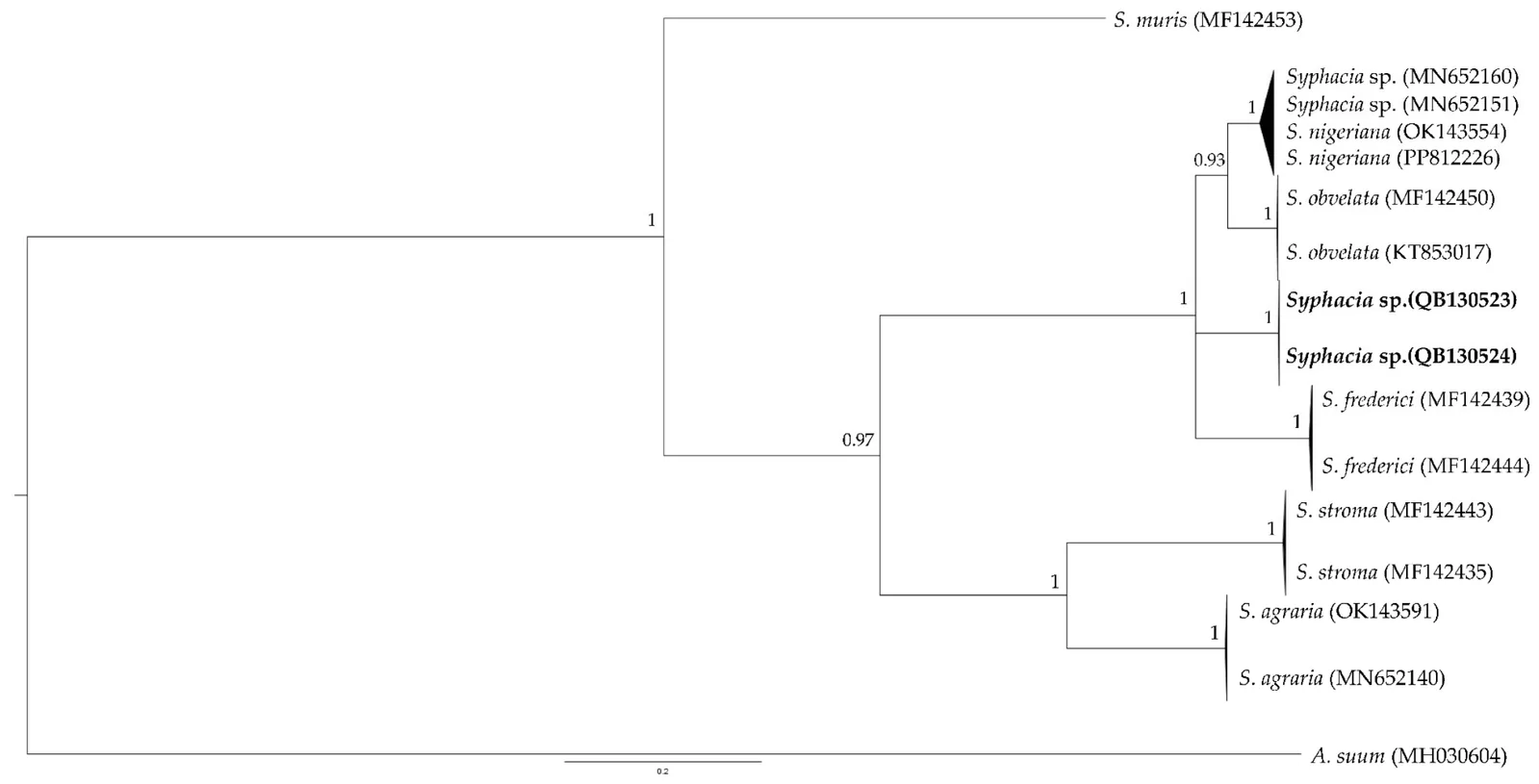
Bayesian inference phylogenetic tree based on sequences of ITS rDNA regions of specimens of *Syphacia* sp. obtained in the present study with respect to the other *Syphacia* spp. sequenced at the same locus previously deposited in GenBank. The analysis was performed using the TIM1+G (G = 0.4251) substitution model. Specimens obtained in this study are marked in bold. *Ascaris suum* was used as an outgroup.

**Figure 8:**
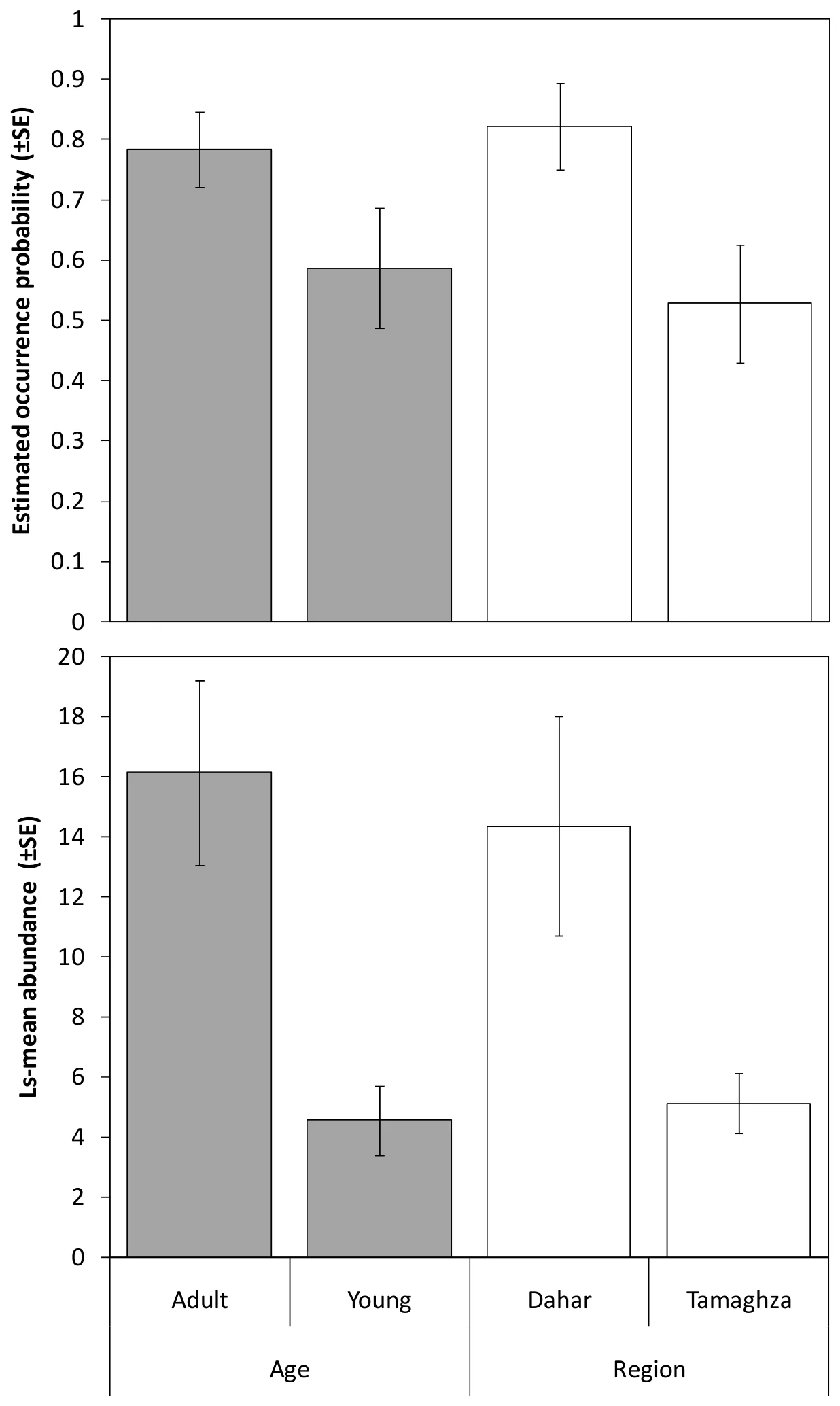
Variation in the occurrence probability and mean abundance of *G. psammomi* according to age and region, as estimated from GLMMs accounting for sex and SMI as other fixed effects and sampling period as a random factor.

**Table 1:** Summary data on the sampled *Psammomys obesus* specimens in the two study areas.

Area	Sample Size					Body Length (mm)		Weight (g)	
	Sex		Age Class		Total	Range	Mean ± SD	Range	Mean ± SD
	Males	Females	<2 Years	>2 Years					
Dahar	30	22	17	35	52	70–195	152.815 ± 22.529	13.2–354.8	139.369 ± 52.023
Tamaghza	41	59	37	63	100	115–195	161.110 ± 21.702	54.4–246.1	148.296 ± 52.022
Total	71	81	54	98	152	70–195	158.272 ± 22.338	13.2–354.8	145.242 ± 52.195

**Table 2:** Descriptive statistics of the occurrence and abundance of the three main recorded parasite species in the study *P. obsesus* sample. The fourth species (*Mesocestoides* sp.) was recorded only in one individual from Dahar, with an abundance of 27 specimens. The variance-to-mean ratio (VMR) largely exceeded 1 for all species, revealing the aggregation of parasites across their hosts.

Parasite Species	Area	Occurrence (%)	Abundance		
			Range	Mean ± SD	VMR
*G. psammomi*	Dahar	44 (84.6%)	0–130	22.308 ± 28.839	37.282
	Tamaghza	53 (53.0%)	0–132	7.190 ± 19.186	51.195
	Total	97 (63.8%)	0–132	12.362 ± 23.958	46.434
*S. aegyptica*	Dahar	19 (36.5%)	0–96	12.711 ± 24.212	46.116
*Syphacia* sp.	Tamaghza	25 (25.0%)	0–646	39.070 ± 115.486	341.361

**Table 3:** Results of GLMMs of the occurrence probability and abundance of the helminth species as functions of sex (female = 1 vs. male = 0), age (adult = 1 vs. young = 0), region (Dahar = 1 vs. Tamaghza = 0), and SMI (scaled mass index; continuous variable) as fixed effects, while accounting for sampling period as a random factor. Occurrence was modeled by considering a binomial distribution with a logit link function, while abundance was modeled using a negative binomial distribution and a log link function. Significant effects are highlighted in bold.

Effect	Occurrence				Abundance			
	*β* ± SE	DF	*t*	*p*	*β* ± SE	DF	*t*	*p*
*G. psammomi*								
Intercept	−1.679 ± 1.479	6	−1.14	0.299	−0.534 ± 1.022	6	−0.52	0.620
Sex	−0.521 ± 0.395	141	−1.32	0.189	−0.464 ± 0.303	141	−1.53	0.128
Age	0.935 ± 0.411	141	2.28	**0.024**	1.266 ± 0.313	141	4.05	**<0.001**
Region	1.412 ± 0.592	141	2.39	**0.018**	1.031 ± 0.316	141	3.26	**0.001**
SMI	0.011 ± 0.010	141	1.08	0.283	0.013 ± 0.007	141	1.73	0.086
*S. aegyptica*								
Intercept	−2.703 ± 1.610	5	−1.680	0.154	−0.873 ± 2.033	5	−0.43	0.685
Sex	0.054 ± 0.666	43	0.080	0.935	0.727 ± 0.853	43	0.85	0.399
Age	0.778 ± 0.748	43	1.040	0.304	0.855 ± 0.892	43	0.96	0.343
SMI	0.008 ± 0.008	43	0.990	0.326	0.015 ± 0.012	43	1.33	0.190
*Syphacia* sp.								
Intercept	1.777 ± 2.423	2	0.730	0.540	3.983 ± 1.783	2	2.230	0.155
Sex	0.023 ± 0.503	94	0.050	0.963	−0.203 ± 0.411	94	−0.490	0.623
Age	0.675 ± 0.533	94	1.270	0.208	0.151 ± 0.418	94	0.360	0.719
SMI	−0.025 ± 0.019	94	−1.330	0.188	−0.022 ± 0.013	94	−1.630	0.106

## Data Availability

Type specimens of the studied helminths were deposited in the “Museu de Ciències Naturals de Barcelona” (Spain) under the following accession numbers: MZB 2025-7284 and MZB 2025-7285 (two specimens of *S. aegyptica*), MZB 2024-3090 to MZB 2024-3093 (four specimens of *G. psammomi*), and MZB 2026-0443 to MZB 2026-0451 (35 males and 35 females of *Syphacia* sp.). Other specimens were deposited in the “Muséum National d’Histoire Naturelle” (Paris, France) under the following accession numbers: MNHN HEL2089 (two specimens of *G. psammomi*); MNHN HEL2088 (one specimen of *S. aegyptica*). Additional specimens of all helminths were deposited in the Jordi Miquel helminth collection at the University of Barcelona (“Secció de Parasitologia, Departament de Biologia, Sanitat i Medi Ambient, Facultat de Farmàcia i Ciències de l’Alimentació”). Three *P. obesus* specimens (skull and carcass) were deposited in the “Museu de Ciències Naturals de Barcelona” (Spain) under the following accession numbers: MZB 2026-0675 to MZB 2026-0677. Two additional *P. obesus* specimens (skull and carcass) were deposited in the “Museu de Ciències Naturals de Granollers” (Spain) under the following accession numbers: MAS004614 and MAS004615.

## Supplementary Materials

The following supporting information can be downloaded at: https://www.mdpi.com/article/10.3390/ani16213492/s1: Table S1. List of sequences used for the Bayesian inference phylogenetic analysis, including *Syphacia* sp. specimens from the present study (in bold) and sequences of *Syphacia* species and *Ascaris suum* (outgroup) retrieved from GenBank, with their corresponding accession numbers, host species, and geographic origins.
