# Supplementary material for: Diversity of Helminth Parasites in the Fat Sand Rat, Psammomys obesus Cretzschmar, 1828, from Tunisia
Source: Animals (Basel). 2026 Nov 4;16(21):3492. doi: 10.3390/ani16213492 (PMC13762355; doi:10.3390/ani16213492)
Supplement: Supplementary file 1 [file animals-16-03492-s001.zip › animals-4582831-supplementary.pdf]

**Table S1.** List of sequences used for the Bayesian inference phylogenetic analysis, including *Syphacia* sp. specimens from the present study (in bold) and sequences of *Syphacia* species and *Ascaris suum* (outgroup) retrieved from GenBank, with their corresponding accession numbers, host species and geographic origins.

| Species | Accession number | Host species | Country |
|---|---|---|---|
| ***Syphacia* sp.** | **QB130523**<br>**QB130524** | ***Psammomys obesus*** | **Tunisia** |
| *Syphacia nigeriana* | PP812226 | *Microtus guentheri* | Turkey |
| *Syphacia nigeriana* | OK143554 | *Microtus arvalis* | Poland |
| *Syphacia obvelata* | MF142450 | *Mus musculus* | Poland |
| *Syphacia obvelata* | KT853017 | *Mus musculus* | India |
| *Syphacia agraria* | MN652140 | *Apodemus agrarius* | Russia |
| *Syphacia agraria* | OK143591 | *Apodemus agrarius* | Poland |
| *Syphacia stroma* | MF142443 | *Apodemus sylvaticus* | Portugal |
| *Syphacia stroma* | MF142435 | *Apodemus sylvaticus* | Ireland |
| *Syphacia frederici* | MF142439 | *Apodemus sylvaticus* | England |
| *Syphacia frederici* | MF142444 | *Apodemus sylvaticus* | Portugal |
| *Syphacia muris* | MF142453 | *Rattus norvegicus* | Netherlands |
| *Syphacia* sp. | MN652151 | *Microtus arvalis* and *Microtus obscurus* hybrid | Russia |
| *Syphacia* sp. | MN652160 | *Microtus arvalis* | Russia |
| *Ascaris suum* | MH030604 | *Sus scrofa domesticus* | USA |
